# Exploring Understanding of Peripheral Artery Disease among Patients at High-Risk in Saudi Arabia: Results from an Interview-Based Study

**DOI:** 10.3390/clinpract14010002

**Published:** 2023-12-21

**Authors:** Sultan Alsheikh, Abdulmajeed Altoijry, Shirin Alokayli, Sarah Ibrahim Alkhalife, Shahad Jamal Alsahil, Hesham AlGhofili

**Affiliations:** 1 Division of Vascular Surgery, Department of Surgery, College of Medicine, King Saud University, Riyadh 11322, Saudi Arabia; aaltoijry@ksu.edu.sa (A.A.); 437201937@student.ksu.edu.sa (S.A.); 438201164@student.ksu.edu.sa (S.I.A.); 438201315@student.ksu.edu.sa (S.J.A.); 2 Vascular Surgery Department, King Salman Heart Center, King Fahad Medical City, Riyadh 11525, Saudi Arabia; halghofili@kfmc.med.sa; 3 Division of Vascular Surgery, Department of Surgery, University of Toronto, Toronto, ON M5S 1A1, Canada

**Keywords:** peripheral arterial disease, atherosclerosis disease, high-risk population, Saudi Arabia, knowledge, interview-based study

## Abstract

Background: The level of awareness of peripheral artery disease (PAD) in Saudi Arabia, especially among populations at high risk, is not currently well known. Therefore, our objective was to assess the existing level of awareness among patients who are at high risk of PAD, as well as their comprehension of the disease. Method: An interview-based cross-sectional study included 1035 participants with risk factors for PAD and collected data on demographics and knowledge domains related to PAD. Results: The statistical analysis was performed using *t*-tests and ANOVA. Overall, participants exhibited poor knowledge, with a mean score of 5.7 out of 26. The highest scores were observed in the risk factor and preventive measure domains, with means of 1.8 out of 7 and 1.8 out of 6, respectively. The factors associated with higher knowledge scores included older age, male gender, higher education, healthcare profession, interviews in vascular settings, previous awareness of PAD, and prior cardio-cerebrovascular interventions. Conclusion: This study underscores the inadequate knowledge of PAD among high-risk individuals. Targeted educational initiatives are essential to bridge this knowledge gap, potentially reducing the burden of PAD-related complications and improving patient outcomes. Efforts should focus on raising awareness about PAD, particularly among high-risk populations.

## 1. Introduction

Peripheral arterial disease (PAD) is a form of systemic atherosclerosis that causes decreased arterial perfusion in the lower extremities and is associated with a significantly higher risk of cardiovascular morbidity and mortality. It is often underdiagnosed and undertreated [1]. PAD is a major global health concern. The prevalence of PAD is 5.6%, affecting approximately 236 million adults worldwide [2]. It represents a growing public health burden, with its prevalence increasing by approximately 45% globally from 2000 to 2015 [2]. In Saudi Arabia, the prevalence of PAD is around 12% [3].

The identification and modification of PAD risk factors have been shown to slow its progression and reduce cardiovascular morbidity and mortality [4]. Patients with peripheral arterial diseases can be asymptomatic for years or rather wait until symptoms worsen. PAD symptoms progress gradually: the patient could have intermittent claudication (IC) pain that changes later into rest pain, and patients may not come for checkups until late stages [3,4]. What makes this a bigger problem is that high-risk patients who already have PAD risk factors in terms of age, smoking, hypertension, and diabetes have a high risk rate for cardiovascular diseases (CVDs) like myocardial infarction and sudden cardiac death. Brain stroke is another factor. Patients with a comorbid history are expected to have a higher PAD risk; hence, regular checkups on their peripheral arteries are crucial [2,3,4].

Understanding the disease risk factors and outcomes greatly impacts the ability to make healthy lifestyle choices and decisions about disease treatment, aiming to reverse adverse outcomes. Managing vascular risk factors and lifestyle changes influences the development and progression of PAD. Being compliant with the medication prescribed by a physician for certain risk factors will reduce the risk of PAD by improving the condition of other risk factors. For example, the use of antihypertensive medication in hypertension patients will improve their blood pressure and their vascular resistance, which directly will reduce PAD risk [5].

Several studies have shown that PAD patients continue to receive suboptimal risk-reducing therapy. Therefore, patients’ good education and knowledge about PAD will hypothetically reduce the risk of PAD-associated complications and enable people to live longer and healthier lives. However, awareness of PAD is low compared to other diseases [5,6]. Limited knowledge about the illness has been recognized as a significant obstacle to modifying its risk factors [6]. Cronin et al. advocated establishing a nationwide awareness campaign for PAD in Ireland in 2015. While some countries have effectively adopted similar strategies, others face obstacles due to financial and infrastructural constraints [7].

Additionally, appointments for patients diagnosed with PAD or those exhibiting risk factors could be optimized for enhanced educational purposes. Little knowledge about PAD in specific populations at high risk of developing PAD necessitates further scrutiny and attention. Therefore, the aim of this study is to assess the existing knowledge of PAD among high-risk individuals. Therefore, we can determine the extent of this knowledge gap and emphasize the importance of risk reduction interventions and educational initiatives to address it. Bridging this knowledge gap is crucial for promoting early detection, appropriate management, and improved outcomes in individuals at high risk for PAD.

## 2. Materials and Methods

This study was performed after approval from the Institutional Review Board of King Saud University College of Medicine, project no. E-22-7566, approved on 8 February 2023.

### 2.1. Study Design and Subject Selection

This cross-sectional, questionnaire-based study was conducted between February 2023 and May 2023. Participants included individuals at high risk of developing PAD, with criteria such as hypertension, dyslipidemia, diabetes, a history of smoking, cerebrovascular disease (CVD), and/or coronary artery disease (CAD). The study took place at King Khalid University Hospital in Riyadh, Saudi Arabia. All eligible patients attending cardiology, neurology, endocrine, nephrology, or vascular clinics, as well as those admitted to these specialty wards with one or more of the mentioned risk factors, were invited to participate to cover a wide range of patients attending different clinics who might exhibit one or more of these risk factors which would be included in our study. With participants who agreed to participate in the study, we specified the location of the interview in terms of whether it was a clinic or ward, and what specialty. A total of 1035 participants were included, with those already diagnosed with PAD excluded from the study.

### 2.2. Data Collection Tool and Process

The survey utilized previously published surveys with the author’s permission [1,8]. Patient interviews were initiated after obtaining their consent and followed a structured format with various sections. These sections covered socio-demographic characteristics such as age, education level, and smoking status. Additionally, participants were queried about their current knowledge of peripheral artery disease (PAD), including awareness, familiarity with the associated symptoms, risk factors, preventive measures, treatment options, and potential complications.

To assess participants’ knowledge, we began the questionnaire with open-ended questions, allowing for spontaneous responses testing for existing knowledge and whether the patients had heard of PAD or had PAD themselves. Multiple-choice options were presented if needed. Importantly, each question included an “I don’t know” option to prevent guessing and ensure an accurate assessment of their existing knowledge.

The interviews, conducted by the authors S. Alokayli, S. Alkhalife, and S. Alsahil, typically lasted between 15 and 20 min. Since Arabic was the native language of our participants, the discussions were conducted in Arabic. The expressions used to inquire about PAD in Arabic can be directly translated into terms such as “peripheral vascular disease”, “arterial intermittent claudication”, “atherosclerosis of the leg’s arteries”, and “arterial blockages in the legs.” An academic English-to-Arabic translator was utilized to select these terms. Before moving to the next section in the questionnaire, the interviewers would make sure the patients understood the term “PAD” to avoid any possible misunderstanding by asking them “Can you please tell me what do you know about peripheral arterial disease?”. Also included was the question “Have you heard about PAD?” in the questionnaire to ensure an adequate understanding/response by the participants.

Each interview concluded with the authors providing concise and reliable information about PAD to spread trustworthy information and education to every participant in the study.

### 2.3. Domains of Knowledge Assessment

Participants underwent an assessment across distinct domains related to PAD, encompassing key aspects of PAD knowledge.

The first domain, focusing on “Symptoms and Signs”, involved evaluating participants’ awareness of various PAD indicators, such as intermittent claudication, rest/night pain, ulcer/gangrene, coolness, paresthesia, and paralysis. In this domain, participants could attain a maximum score of 6, with one point allocated for each correctly identified symptom or sign.

The second domain, “Risk Factors”, involved participants being queried about the recognized risk factors associated with PAD. These included old age, smoking, diabetes, hypertension, dyslipidemia, male gender, and personal or family history of atherosclerosis. A maximum score of 7 points was attainable in this domain, with one point awarded for each correctly identified risk factor.

The third domain, “Preventive Measures”, delved into participants’ knowledge of strategies to prevent PAD. This included smoking cessation, dietary modifications, lifestyle adjustments, diabetes management, hypertension control, and the use of risk reduction medications such as statins or aspirin. In this domain, participants could achieve a maximum score of 6 points.

In the fourth domain, “Management Options”, participants were questioned about their understanding of the available treatment approaches for PAD, encompassing medical management only, surgical management only, or a combination of both. In this domain, a maximum score of 3 points was possible.

Lastly, the fifth domain focused on “Complications”, exploring participants’ awareness of the potential adverse outcomes associated with PAD. These complications encompassed minor tissue loss (e.g., non-healing ulcers or toe gangrene), major tissue loss (e.g., limb loss below or above the knee), cardiac complications, cerebrovascular complications, and mortality. In this domain, participants could earn a maximum score of 5 points.

The overall knowledge score for each participant was calculated by summing their scores across these five domains, with the highest achievable score being 26. This comprehensive assessment allowed for a thorough evaluation of each participant’s understanding of PAD and its various facets.

### 2.4. Statistical Analysis

The data obtained were analyzed using the Statistical Package for the Social Sciences for Windows (version 22.0; IBM Corp., Armonk, NY, USA). The data are presented as frequencies and percentages for nominal variables and as means ± standard deviations for numerical variables. Each correctly identified item in the scores received one point. We conducted *t*-tests to compare means between two groups and ANOVA for comparisons involving three or more groups. Statistical significance was considered at *p*-values less than 0.05 for each analysis.

## 3. Results

A total of 1035 participants were interviewed, with 52.3% of them being female. The mean age of participants was 50.7 ± 14.7 years, and 72.2% had a high school education or higher. The majority of participants (93.5%) did not work in a healthcare occupation. Only 32.6% of participants had heard about PAD before. Refer to Table 1 for further details.

Participants displayed generally poor knowledge, with an overall knowledge mean of 5.7 ± 6.3 out of a possible 26. Specifically, their knowledge about complications was notably deficient, scoring a mean of 0.7 ± 1.2 out of 5. Participants exhibited a somewhat higher but still limited level of understanding in the “Symptoms and Signs” domain, averaging 0.9 ± 1.4 out of 6. The highest knowledge score was observed in the “Risk Factors” and “Preventive Measures” domains with 1.8 ± 1.9 out of 7 and 1.8 ± 2 out of 6, respectively.

Table 2 shows the percentages of participants who exhibited awareness of PAD and their corresponding ability to accurately identify specific PAD characteristics within the five knowledge domains assessed. Notably, 28.2% of participants correctly identified numbness as a symptom of PAD. Conversely, only 7.7% recognized non-healing ulcer/gangrene as a symptom, while a substantial 89.1% were unaware that intermittent claudication could be indicative of PAD. Regarding the risk factors, approximately half of the participants (48.4% and 47.9%) correctly identified diabetes and hypertension.

However, a considerable 90.5% were uninformed that the risk of PAD could be heightened with a personal or family history of atherosclerosis. Additionally, only 19.1% acknowledged smoking as a risk factor for PAD. Knowledge of preventive measures was generally low, with smoking cessation being the least recognized, garnering only 0.9% recognition. Participants demonstrated limited awareness of complications. Minor and major amputation as complications were recognized by 10.6% and 18.9% of participants, respectively. Furthermore, only 8.4% acknowledged mortality as a potential complication associated with PAD. The aggregate percentage of correct responses among participants was found to be 19.6%.

**Figure 1:**
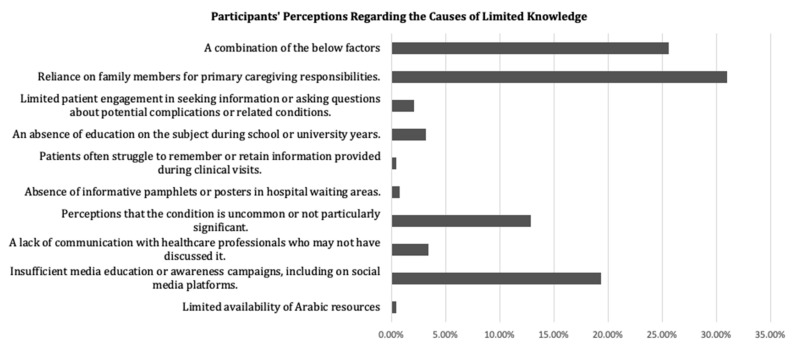
Participants’ perceptions regarding the causes of limited knowledge.

As shown in Table 3, participants demonstrating a higher knowledge score regarding PAD exhibited certain characteristics. These included being aged 50 years or older (6.4 ± 6.6 vs. 5 ± 5.9; *p* = 0.004), being male (6.6 ± 6.7 vs. 4.9 ± 5.9; *p* < 0.001), possessing a university-level education (7.2 ± 6.8; *p* < 0.001), being a physician (15.4 ± 7.1; *p* < 0.001), being interviewed in a vascular ward or clinic (9.5 ± 7.6; *p* < 0.001), being an ex-smoker (9.5 ± 7; *p* < 0.001), having heard/read about PAD (11.6 ± 6.2; *p* < 0.001), and having undergone prior cardio- or cerebrovascular interventions (7.3 ± 6.7; *p* = 0.024). These factors were associated with higher knowledge scores in the context of PAD. Participants provided qualitative responses regarding the factors contributing to limited knowledge. Figure 1 depicts the participants’ perceptions of the underlying causes of this limitation.

## 4. Discussion

### 4.1. Findings and Comparison with Existing Literature

Atherosclerosis stands as the leading cause of mortality in Saudi Arabia [9]. PAD prevalence is on the rise globally in both developed and developing nations [10]. Among Saudis aged over 44, 12% are affected, two-thirds of whom have at least two risk factors. This number increases to around 25% with additional risk factors [10]. Alarmingly, up to half of all individuals with PAD remain undiagnosed [4].

Despite the lack of established benchmarks for acceptable diagnosis awareness rates among patients or the general public, our study revealed a notably lower awareness of PAD compared to other conditions. Among high-risk patients, 67.4% had never even heard of the disease, a stark contrast to the 22% unfamiliarity with stroke in Saudi Arabia [11]. Similar trends were observed in surveys conducted on the general population in Canada and the United States [12,13].

Intermittent claudication is the predominant symptom associated with PAD [4]. Approximately one-third of PAD patients exhibit this symptom [4]. However, only 10% of participants in our study recognized intermittent claudication, while rest/night pain and tissue loss were identified by 12% and 7.7%, respectively. That could be explained by the patient experience. IC is not usually addressed as a significant symptom from the patient’s perspective and they most likely will not seek medical counseling for it, but when it becomes worse and the patient starts to suffer from more ischemic chronic symptoms such as rest pain, for example, they are most likely to seek help to relieve the pain. Also, taking into consideration the different pain thresholds for patients, some wait for too long to seek a medical opinion. In comparison, 80.5% of the Saudi general population recognized chest pain as a symptom of CAD [14]. In a different study, approximately half of the participants recognized acute sensorimotor deficits and about 60% identified slurred speech as clinical signs of a stroke [15].

Inadequate awareness about PAD can be attributed to various factors, including suboptimal knowledge among general practitioners (GPs) [1]. Hence, it is possible that GPs’ education regarding the disease is insufficient. Our data corroborate this observation, as patients interviewed in vascular surgery settings exhibited significantly superior knowledge about the disease compared to those interviewed in other settings.

Additionally, the media focus on CAD and CVD leaves sparse information about PAD, exacerbating the knowledge gap. Charasson et al. demonstrated the impact of widely disseminated information about CAD and CVD among physicians. Their study revealed that 48% of GPs answered questions correctly on CAD, 3% on CVD, and only 0.4% in PAD cases [16]. The influence of media may extend to patients as well, potentially resulting in limited awareness about PAD among the general population.

Hypertension, diabetes, and dyslipidemia are established risk factors for PAD and its complications. These conditions are highly prevalent among the Saudi population, with each affecting up to approximately 70% of retired patients in primary care settings [17]. In our study, 48.4% recognized diabetes, 47.9% identified hypertension, and 28.2% acknowledged hyperlipidemia as risk factors for the disease. Similar results were reported in a systematic review that analyzed the findings of six studies among the general population [18].

Only 19.6% identified themselves as current or ex-smokers, and overall, only 19.1% identified smoking as a risk factor. This variable in our study might be influenced by reporting reluctance due to social stigma, a phenomenon well documented in conditions like lung cancer [19]. Interestingly, only 0.9% of participants considered smoking cessation as a preventive measure, indicating a lack of awareness about the relationship between smoking and PAD. Earlier studies have indicated that some ex-smokers either consistently denied the link between smoking and PAD or needed ongoing reassurance and motivation to sustain their non-smoking status [18,20].

Furthermore, knowledge about PAD consequences was profoundly lacking among high-risk patients. Many were unaware of the elevated risks of myocardial infarction, stroke, and death associated with PAD. The denial of the severity of the disease, a lack of education, or a combination of both factors could account for these results.

### 4.2. Clinical Implications and Areas of Improvement

Education plays a vital role in the early detection, presentation, and treatment of PAD [12]. A study conducted in Toronto demonstrated the effectiveness of education [21]. In this study, patients were surveyed, educated, and later re-engaged after a few weeks [21]. Deficiencies in patient knowledge about PAD may lead to behaviors resulting in poor clinical outcomes. Studies have shown that patients’ perceptions of their personal risk of adverse events and the effectiveness of preventive therapy influence their adoption of preventive health behaviors [22]. The increasing availability of patient-targeted healthcare information and patients’ desire to participate in healthcare decisions highlight the growing role of patients in perceiving preventive practices [22]. Therefore, patient factors can significantly impact the extent to which risk factors are mitigated in PAD.

Our study identified a group of patients reliant on family members for care. This group demonstrated a passive approach to seeking information, lacking the necessary knowledge to actively participate in their own healthcare decisions, or sometimes, not being encouraged to [20].

Health literacy refers to the extent to which individuals possess the capability to locate, comprehend, and apply information to make informed decisions and take actions related to their health. Effective education strategies should consider functional health literacy, employ patient teach-back methods, and utilize patient-preferred formats to bridge knowledge gaps. Given that PAD is a chronic, costly, and incurable disease influenced by behavior and lifestyle, education interventions are crucial to empowering patients with knowledge, improving their competence, confidence, and ability to self-manage the disease, and facilitating engagement with healthcare providers [23,24,25]. Enhancing patient knowledge using educational interventions has been linked to greater self-efficacy in managing various chronic and incurable conditions [26].

Moreover, educating physicians, especially those caring for patients at high risk of PAD, is imperative. Our study revealed that patients interviewed in vascular clinic settings exhibited better knowledge about the disease and its implications. National awareness campaigns, such as the one implemented by the National Heart, Lung, and Blood Institute (NHLBI) in the USA, offer valuable resources and events to enhance PAD knowledge among the public and healthcare providers [27].

Leveraging online platforms and social media can further enhance the reach of educational efforts, as demonstrated by successful campaigns like the Act Fast initiative in the United Kingdom, which utilized TV adverts and national media coverage to enhance stroke awareness. Dombrowski et al. found that the campaign resulted in significantly heightened stroke awareness among the public [28]. Similarly, Flynn et al. observed a noticeable increase in information-seeking behavior [29].

To further advance our understanding, future research should focus on evaluating the best practices and effective strategies to improve knowledge among the general population and patients at high risk of PAD, ensuring comprehensive and tailored educational interventions that empower individuals to manage their condition effectively.

### 4.3. Limitations and Strengths

This project is an important milestone specifically addressing the knowledge of PAD among high-risk populations in Saudi Arabia, boasting a large sample with over 1000 responses. Furthermore, the data collection employed an interview-based design and open-ended questions, effectively limiting the impact of information bias on study results and minimizing guessing by participants. Additionally, data were consistently collected by the same research team to ensure the accuracy of the collected data.

Moreover, this study could be a part of a community-based initiative to raise PAD awareness among Saudi high-risk populations, and its results could provide an additional motive for physicians to address the gaps in knowledge during clinical practice. However, it is important to acknowledge the limitations of this study when interpreting its findings. Firstly, this study was conducted at a single center, raising concerns about the generalizability of our data to the broader population. Secondly, there were apparent misunderstandings concerning the distinctions between PAD and common conditions like musculoskeletal disorders, as well as misconceptions about what is considered normal within the aging process. This confusion was exacerbated by a lack of basic understanding of PAD’s pathophysiology among the surveyed subjects, potentially hindering their ability to recognize associated risk factors.

In an effort to address these challenges, our team employed various verified medical terminologies in Arabic while posing the central study question (“Have you heard about peripheral artery disease?”). These terms encompass PAD, peripheral vascular disease, arterial intermittent claudication, atherosclerosis of the leg’s arteries, and arterial blockages in the legs. However, it is important to note that PAD is commonly referred to using multiple terms by both patients and healthcare practitioners, introducing further uncertainty. This limitation aligns with similar challenges reported in previous studies related to PAD [12,18].

## 5. Conclusions

In conclusion, our study has identified a notable need for more knowledge pertaining to PAD among high-risk individuals within our specific study settings. Remarkably, patients who had prior exposure to information regarding PAD exhibited statistically significant improvements in their knowledge scores. This trend was consistently observed among patients interviewed in vascular surgery settings compared to those in alternative clinical settings. We anticipate that educating patients and their families during clinical encounters will yield profound benefits. Our findings emphasize the critical role of educational interventions during clinical encounters, with the ultimate expectation of diminishing the need for PAD-related amputation and mortality rates.

We see more education for other conditions like coronary artery disease, which is certainly a global health problem that has been addressed over the years, with adequate education for the general population as well as high-risk patients. Hence, heart diseases are well known, and patients would agree on the importance of lifestyle modification as a main element to avoid further deterioration in the condition or aid in primary prevention of the disease. Such education is required for PAD, which also carries a high morbidity and mortality risk.

Future research is advised to explore the most effective educational methodologies. The proposed strategies can focus on promoting a proactive management approach using targeted interventions. These interventions might encompass continuous medical education programs, public awareness campaigns, and diverse educational outreach initiatives. Also, in searching for what might stop patients from seeking medical opinions, this could be lack of awareness of diseases, bearable symptoms, financial reasons, or a lack of facilities in the local area. Regardless, PAD is a worldwide problem that needs more focus to improve patients’ health, support lifestyle modifications, and have a positive impact on hospital economies.

## Figures and Tables

**Table 1:** Demographics.

Variable	Mean ± SD, N (%)
Age (years)	50.7 ± 14.7
Sex	
Male	493 (47.7)
Female	541 (52.3)
Level of Education	
No formal education	128 (12.4)
Primary school	83 (8)
Intermediate school	77 (7.5)
High school	192 (18.6)
University	554 (53.6)
Medical background	
Non-healthcare	967 (93.5)
Healthcare but were not physicians	59 (5.7)
Physicians	8 (0.8)
Smoking status	
Never smoked	831 (80.4)
Ex-smoker	83 (8)
Current smoker	120 (11.6)
Have you heard about PAD? (yes)	337 (32.6)

PAD, peripheral artery disease.

**Table 2:** Proportion of PAD-aware participants that correctly identified specific PAD characteristics.

Knowledge Domain	N (%)
Symptoms and Signs	
Intermittent claudication	104 (10.1)
Rest/night pain	123 (12)
Ulcer/gangrene	79 (7.7)
Coolness	93 (9)
Numbness	290 (28.2)
Paralysis	205 (20)
Risk Factors	
Old age	178 (17.3)
Smoking	196 (19.1)
Diabetes	497 (48.4)
Hypertension	492 (47.9)
Hyperlipidaemia	290 (28.2)
Male gender	38 (3.7)
Personal/family history	98 (9.5)
Preventive Measures	
Smoking cessation	9 (0.9)
Dietary changes	462 (45)
Lifestyle changes	419 (40.8)
Diabetes control	380 (37)
Hypertension control	310 (30.2)
Medications (risk reduction therapy)	84 (8.2)
Management Options	
Medical treatment (including lifestyle changes and exercise)	101 (9.8)
Surgical	86 (8.4)
Both	171 (16.7)
Complications	
Tissue loss/gangrene (minor tissue loss)	109 (10.6)
Limb or part of limb loss (major tissue loss)	194 (18.9)
Myocardial infarction	239 (23.3)
Stroke	134 (13)
Mortality	86 (8.4)

**Table 3:** Knowledge score by participant demographics.

Variable	Mean ± SD, N (%)	95% CI	t-Value (df)/F	*p*-Value
Age (years)				
≥50	6.4 ± 6.6	[0.65, 2.19]	3.6 (952.9)	0.004
<50	5 ± 5.9			
Sex				
Male	6.6 ± 6.7	[0.92, 2.46]	4.3 (985.1)	<0.001
Female	4.9 ± 5.9			
Level of education				
No formal education	2.35 ± 4.1		22.2	<0.001 *
Primary school	3.4 ± 4.9			
Intermediate school	4.2 ± 5.5			
High school	5.2 ± 5.6			
University	7.2 ± 6.8			
Medical background				
Non-healthcare	5.3 ± 6		38.3	<0.001 *
Healthcare but not a physician	11.4 ± 8			
Physician	15.4 ± 7.1			
Interview place				
Cardiology ward/clinic	4.8 ± 5.7		12.2	<0.001 *
Endocrine ward/clinic	5.3 ± 6			
Neurology ward/clinic	4.6 ± 5.6			
Nephrology ward/clinic	4.7 ± 5.7			
Vascular ward/clinic	9.5 ± 7.6			
Smoking status				
Never smoked	5.4 ± 6.3		16.9	<0.001 *
Ex-smoker	9.5 ± 7			
Current smoker	5 ± 5.5			
Heard about PAD?				
Yes	11.6 ± 6.2	[8.07, 9.52]	23.91 (477.5)	<0.001
No	2.8 ± 4			
Previous cardiocerebrovascular interventions				
Yes	7.3 ± 6.7	[0.91, 2.97]	3.7 (281.7)	0.024
No	5.3 ± 6.2			

* ANOVA test.

## Data Availability

The data presented in this study are available upon request from the corresponding authors.

## References

[1] Alsheikh S., AlGhofili H., Alayed O.A., Aldrak A., Iqbal K., Altoijry A. (2022). Are primary care physicians aware of peripheral artery disease risk reduction and management in the Saudi healthcare transformation era? A health cluster observational study. Vascular.

[2] Aday A.W., Matsushita K. (2021). Epidemiology of Peripheral Artery Disease and Polyvascular Disease. Circ. Res..

[3] Al-Sheikh S.O., Aljabri B.A., Al-Ansary L.A., Al-Khayal L.A., Al-Salman M.M., Al-Omran M.A. (2007). Prevalence of and risk factors for peripheral arterial disease in Saudi Arabia. A pilot cross-sectional study. Saudi Med. J..

[4] Smith S.C., Benjamin E.J., Bonow R.O., Braun L.T., Creager M.A., Franklin B.A., Gibbons R.J., Grundy S.M., Hiratzka L.F., Jones D.W. (2011). AHA/ACCF Secondary Prevention and Risk Reduction Therapy for Patients with Coronary and other Atherosclerotic Vascular Disease: 2011 update: A guideline from the American Heart Association and American College of Cardiology Foundation. Circulation.

[5] Chen D.C., Armstrong E.J., Singh G.D., Amsterdam E.A., Laird J.R. (2015). Adherence to guideline-recommended therapies among patients with diverse manifestations of vascular disease. Vasc. Health Risk Manag..

[6] Keelan S., Foley N., Healy D., Kheirelseid E., McHugh S., Moneley D., Naughton P. (2022). Poor patient awareness of peripheral arterial disease, it is time to optimize the clinical visit. Surgeon.

[7] Cronin C.T., McCartan D.P., McMonagle M., Cross K.S., Dowdall J.F. (2015). Peripheral artery disease: A marked lack of awareness in Ireland. Eur. J. Vasc. Endovasc. Surg..

[8] Mubaraki A.A., Alqahtani A.S., Almalki A.A., Almalki A.H., Alamri H.M., Aburass M.K., Althumali Z.H. (2021). Public knowledge and awareness of stroke among adult population in Taif city, Saudi Arabia. Neurosciences.

[9] World Health Organization, Saudi Arabia Health Profile 2015. https://applications.emro.who.int/dsaf/EMROPUB_2016_EN_19272.pdf?ua=1&ua=1.2015.

[10] Fowkes F.G., Rudan D., Rudan I., Aboyans V., Denenberg J.O., McDermott M.M., Norman P.E., Sampson U.K., Williams L.J., Mensah G.A. (2013). Comparison of global estimates of prevalence and risk factors for peripheral artery disease in 2000 and 2010: A systematic review and analysis. Lancet.

[11] Alzahrani H.A., Wang D., Bakhotmah B.A., Hu F.B. (2014). Risk factors for peripheral artery disease among patients with diabetes in Saudi Arabia. Vasc. Med..

[12] Alaqeel A., AlAmmari A., AlSyefi N., Al-Hussain F., Mohammad Y. (2014). Stroke awareness in the Saudi community living in Riyadh: Prompt public health measures must be implemented. J. Stroke Cerebrovasc. Dis..

[13] Hirsch A.T., Murphy T.P., Lovell M.B., Twillman G., Treat-Jacobson D., Harwood E.M., Mohler E.R., Creager M.A., Hobson R.W., Robertson R.M. (2007). Gaps in public knowledge of peripheral arterial disease: The first national PAD public awareness survey. Circulation.

[14] Lovell M., Harris K., Forbes T., Twillman G., Abramson B., Criqui M.H., Schroeder P., Mohler E.R., Hirsch A.T. (2009). Peripheral arterial disease: Lack of awareness in Canada. Can. J. Cardiol..

[15] Al Harbi K.M., Alluhidan W.A., Almatroudi M.I., Almuhanna N.I., Alotaibi N.M. (2022). Knowledge and Attitude of General People towards Symptoms of Heart Attack and the Impact of Delay Time in Riyadh, Saudi Arabia. Cureus.

[16] Charasson M., Mahe G., Le Brun C., Jaquinandi V., Rossignol E., Le Faucheur A., Omarjee L. (2018). Atherosclerosis knowledge—Diagnosis and management in primary care. Vasa.

[17] Al Turki Y.A. (2014). Cardiovascular risk factors among retired attendees visiting primary care clinics. Pak. J. Med. Sci..

[18] Bridgwood B.M., Nickinson A.T., Houghton J.S., Pepper C.J., Sayers R.D. (2020). Knowledge of peripheral artery disease: What do the public, healthcare practitioners, and trainees know?. Vasc. Med..

[19] Ostroff J.S., Banerjee S.C., Lynch K., Shen M.J., Williamson T.J., Haque N., Riley K., Hamann H.A., Rigney M., Park B. (2022). Reducing stigma triggered by assessing smoking status among patients diagnosed with lung cancer: De-stigmatizing do and don’t lessons learned from qualitative interviews. PEC Innov..

[20] Wann-Hansson C., Wennick A. (2016). How do patients with peripheral arterial disease communicate their knowledge about their illness and treatments? A qualitative descriptive study. BMC Nurs..

[21] El Morr C., AlHamzah M., Ng P., Purewal A., Al-Omran M. (2017). Knowledge of peripheral arterial disease: Results of an intervention to measure and improve PAD knowledge in Toronto. Vascular.

[22] Bridgwood B., Lager K.E., Mistri A.K., Khunti K., Wilson A.D., Modi P. (2018). Interventions for improving modifiable risk factor control in the secondary prevention of stroke. Cochrane Database Syst. Rev..

[23] Scully R.E., Arnaoutakis D.J., DeBord Smith A., Semel M., Nguyen L.L. (2018). Estimated annual health care expenditures in individuals with peripheral arterial disease. J. Vasc. Surg..

[24] Murphy T.P., Cutlip D.E., Regensteiner J.G., Mohler E.R., Cohen D.J., Reynolds M.R., Massaro J.M., Lewis B.A., Cerezo J., Oldenburg N.C. (2015). Supervised exercise, stent revascularization, or medical therapy for claudication due to aortoiliac peripheral artery disease: The CLEVER study. J. Am. Coll. Cardiol..

[25] Abaraogu U.O., Ezenwankwo E.F., Dall P.M., Seenan C.A. (2018). Living a burdensome and demanding life: A qualitative systematic review of the patients experiences of peripheral arterial disease. PLoS ONE.

[26] Ludman E.J., Peterson D., Katon W.J., Lin E.H., Von Korff M., Ciechanowski P., Young B., Gensichen J. (2013). Improving confidence for self care in patients with depression and chronic illnesses. Behav. Med..

[27] Castaneda P., Sales A., Osborne N.H., Corriere M.A. (2019). Scope, Themes, and Medical Accuracy of eHealth Peripheral Artery Disease Community Forums. Ann. Vasc. Surg..

[28] Dombrowski S.U., Mackintosh J.E., Sniehotta F.F., Araujo-Soares V., Rodgers H., Thomson R.G., Murtagh M.J., Ford G.A., Eccles M.P., White M. (2013). The impact of the UK ‘Act FAST’ stroke awareness campaign: Content analysis of patients, witness and primary care clinicians’ perceptions. BMC Public Health.

[29] Flynn D., Ford G.A., Rodgers H., Price C., Steen N., Thomson R.G. (2014). A time series evaluation of the FAST National Stroke Awareness Campaign in England. PLoS ONE.

